# The total and direct effects of systolic and diastolic blood pressure on cardiovascular disease and longevity using Mendelian randomisation

**DOI:** 10.1038/s41598-021-00895-2

**Published:** 2021-11-08

**Authors:** Io Ieong Chan, Man Ki Kwok, C. Mary Schooling

**Affiliations:** 1 School of Public Health, Li Ka Shing Faculty of Medicine, The University of Hong Kong, 7 Sassoon Road, Pokfulam, Hong Kong SAR China; 2 Graduate School of Public Health and Health Policy, City University of New York, New York, USA

**Keywords:** Cardiovascular diseases, Genetics research, Risk factors, Epidemiology

## Abstract

The 2017 American College of Cardiology/American Heart Association (ACC/AHA) blood pressure (BP) guidelines lowered the hypertension threshold to ≥ 130/80 mmHg, but the role of diastolic BP remains contested. This two-sample mendelian randomisation study used replicated genetic variants predicting systolic and diastolic BP applied to the UK Biobank and large genetic consortia, including of cardiovascular diseases and parental lifespan, to obtain total and direct effects. Systolic and diastolic BP had positive total effects on CVD (odds ratio (OR) per standard deviation 2.15, 95% confidence interval (CI) 1.95, 2.37 and OR 1.91, 95% CI 1.73, 2.11, respectively). Direct effects were similar for systolic BP (OR 1.83, 95% CI 1.48, 2.25) but completely attenuated for diastolic BP (1.18, 95% CI 0.97, 1.44), although diastolic BP was associated with coronary artery disease (OR 1.24, 95% CI 1.03, 1.50). Systolic and diastolic BP had similarly negative total (− 0.20 parental attained age z-score, 95% CI − 0.22, − 0.17 and − 0.17, 95% CI − 0.20, − 0.15, respectively) and direct negative effects on longevity. Our findings suggest systolic BP has larger direct effects than diastolic BP on CVD, but both have negative effects (total and direct) on longevity, supporting the 2017 ACC/AHA guidelines lowering both BP targets.

## Introduction

Elevated blood pressure (BP), or hypertension, increases the risk of dying from cardiovascular disease (CVD)^1^, and is one of the most important factors determining population health worldwide^2^. The number of adults with hypertension has increased from 594 million in 1975 to 1.13 billion in 2015, driven by increasing prevalence in south Asia and sub-Saharan Africa^3^, as well as the evolving definition of hypertension^4^. In 2017 the American College of Cardiology/American Heart Association (ACC/AHA) guidelines recommended reducing the threshold for hypertension from 140/90 to 130/80 mmHg^5,6^, largely based on results from the Systolic Blood Pressure Intervention Trial (SPRINT). However, the SPRINT trial focused on systolic BP, which raised the questions as to whether lowering the diastolic BP threshold was necessary^7^, and reignited interest in the importance of BP components, particularly diastolic BP^8,9^.

Trials of specifically reducing diastolic BP without changing systolic BP are difficult to conduct, meaning the importance of diastolic BP remains to be fully elucidated. Observational studies are open to both confounding and selection bias which may bias estimates. Mendelian randomisation (MR) using genetic variants as instrumental variables^10^, which are randomly allocated at conception, is much less open to confounding^11^. Previous MR studies suggested diastolic BP was not associated with CVD overall or by subtype independent of systolic BP^12^, and was inversely associated with risk of large artery stroke^13^, suggesting some cerebrovascular-protective effects of diastolic BP. Another MR study suggested diastolic BP was more strongly associated with shorter lifespan than systolic BP^14^, but did not consider the relative contribution of systolic and diastolic BP together, i.e. direct as well as total effects. From a population health perspective, the overall health benefits of achieving a low diastolic BP is probably more relevant because elevated BP likely has other non-cardiovascular consequences^15^.

Here, we examined the effects of systolic and diastolic BP on CVD, its subtypes, and lifespan, using multivariable MR, which is an increasingly popular method of considering multiple risk factors simultaneously^16,17^. Specifically, we used replicated genetic instruments and the largest publicly available genetic studies of CVD and longevity.

## Results

### Total effects of systolic and diastolic BP

The 272 and 267 strong, independent and replicated SNPs predicting systolic and diastolic BP had mean (range) F-statistic for systolic and diastolic BP of 83.2 (29.3–612.4) and 90.7 (30.0–818.1), indicating bias due to weak instruments is unlikely. These SNPs explained approximately 2.59 and 2.96% of the variance of systolic and diastolic BP, respectively. Of these SNPs, 10 SNPs for systolic BP and 9 SNPs for diastolic BP were not available in the parental lifespan GWAS, nor were their proxies. The I^2^ statistics were at least 92.5% for systolic, and 93.8% for diastolic BP. Power calculations showed that at 5% alpha, this study has 80% power to detect, in the univariable MR. an odds ratio (OR) of about 1.11 for coronary artery disease, 1.11 for ischaemic stroke (1.19 in the UK Biobank), 1.09 for heart failure, 1.08 for atrial fibrillation and a beta coefficient of 0.04 standardised combined parental attained age. Supplementary Tables S1-S2 show the SNPs used. These SNPs explained a larger proportion of the phenotypic variance of systolic and diastolic BP, than of the outcomes considered, suggesting reverse causality was unlikely.

**Figure 1:**
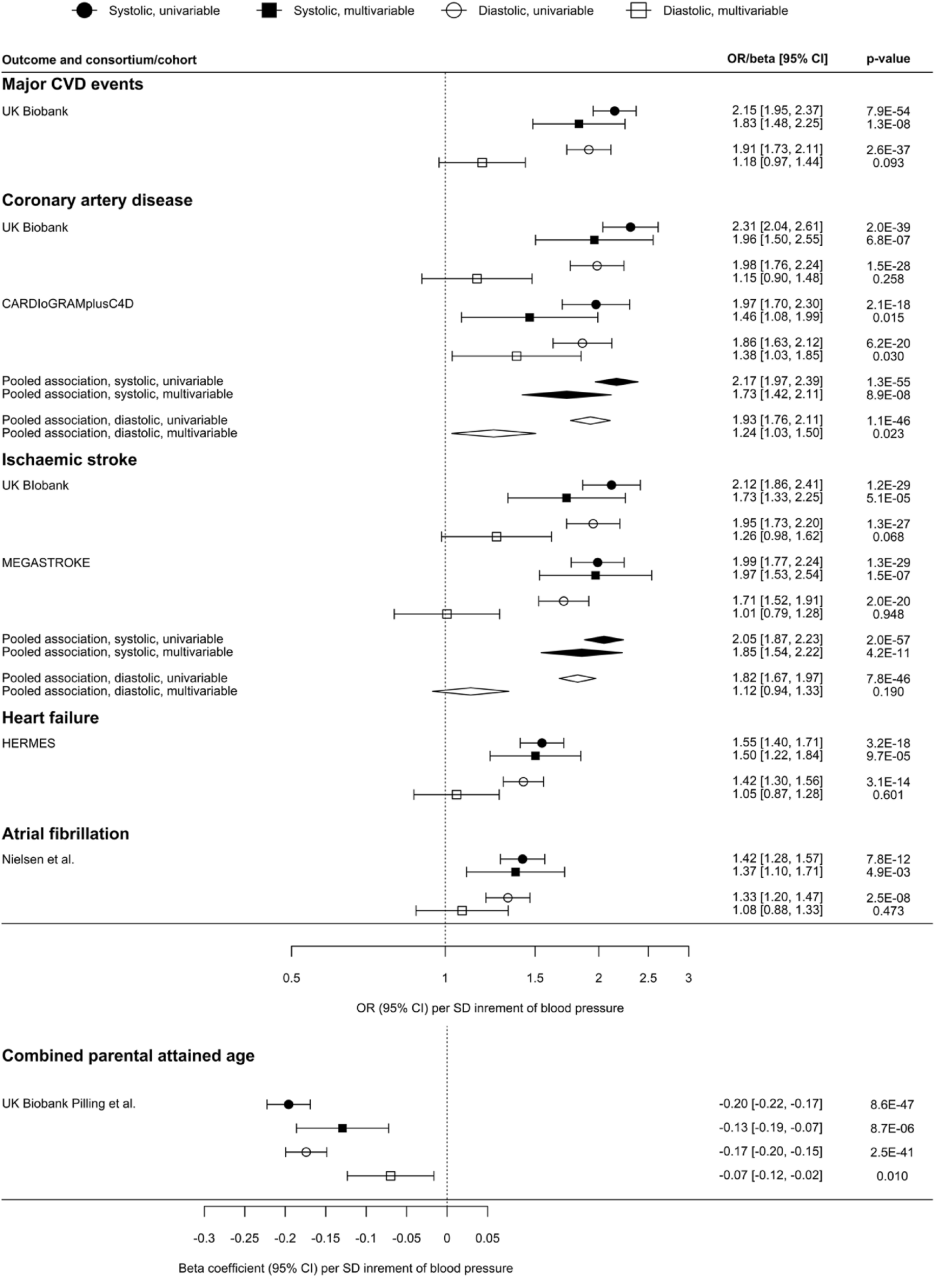
Univariable and multivariable Mendelian randomisation estimates of the total and direct effects of systolic and diastolic blood pressure on cardiovascular disease, and longevity based on parental lifespan.

Both systolic and diastolic BP were positively associated with major CVD events, (OR 2.15 [95% confidence interval (CI) 1.95, 2.37] and 1.91 [95% CI 1.73, 2.11], respectively) (Fig. 1 and supplementary Table S3). For CVD subtypes, both systolic and diastolic BP were most strongly associated with coronary artery disease, and least strongly associated with atrial fibrillation, as well as being positively associated with ischaemic stroke and heart failure. The MR-Egger intercepts (minimum P for intercept 0.081) did not suggest these IVW estimates were invalid. Higher systolic (beta coefficient − 0.20 [95% CI − 0.22, − 0.17]) and diastolic (beta coefficient − 0.17 [95% CI − 0.20, − 0.15]) BP were inversely associated with parental lifespan (Fig. 1 and supplementary Table S3), equivalent to 2.9 and 2.6 years lower combined parental attained age per SD of BP. Estimates obtained using the weighted median and MR-PRESSO were generally concordant in direction and magnitude. Using Phenoscanner, we identified 15 and 18 potential pleiotropic SNPs for systolic and diastolic BP that were strongly (P < 5 × 10^–8^) associated with mostly BMI (supplementary Tables S4A and B). Excluding these SNPs gave similar MR estimates (supplementary Table S5). Higher pulse pressure was positively associated with all CVD outcomes considered, and inversely associated with parental lifespan (supplementary Table S6).

### Direct effects of systolic and diastolic BP

Of the 392 SNPs for systolic and diastolic BP, 312 SNPs were independent (r^2^ < 0.001), rs73033340 or its proxies were not available in the systolic BP GWAS, leaving 311 SNPs (299 for parental lifespan) for the multivariable MR analyses. Of these, 178 SNPs were associated with both systolic (243) and diastolic BP (246) at P < 5 × 10^–8^. The multivariate F-statistics for the genetic instruments for systolic and diastolic BP, taking into account their phenotypic correlation, were 31.1 and 32.1, respectively, indicating strong instruments for multivariable MR.

Direct effects of systolic and diastolic BP were attenuated compared to the total effects, with stronger associations for systolic than diastolic BP (Fig. 1 and supplementary Table S7). Systolic (OR 1.83 [95% CI 1.48, 2.25]), but not diastolic BP (OR 1.18 [95% CI 0.97, 1.44]) remained positively associated with major CVD events. Systolic BP was positively associated with all CVD subtypes considered. In contrast, diastolic BP remained only nominally associated with coronary artery disease (OR 1.24 [95% CI 1.03, 1.50], p = 0.023), but not with ischaemic stroke (OR 1.12 [95% 0.94, 1.33], heart failure (OR 1.05 [95% CI 0.87, 1.28]) or atrial fibrillation (OR 1.08 [95% CI 0.88, 1.33]). Higher systolic BP (beta coefficient − 0.13 [95% CI − 0.19, − 0.07] and diastolic BP (beta coefficient − 0.07 [95% CI − 0.12, − 0.02]) remained inversely associated with parental lifespan (Fig. 1 and supplementary Table S7), equivalent to − 1.9 and − 1.0 years per SD BP, respectively. There was statistical evidence of pleiotropic effects from the modified Cochran’s Q test, and potential directional pleiotropy based on the multivariable MR-Egger intercepts for systolic BP and the UK Biobank estimates including major CVD events (p for intercept = 0.03) and coronary artery disease (p for intercept = 0.03). However, the MR-Egger estimates suggested similar but larger effect sizes (supplementary Table S7), and estimates from other multivariable MR methods, as well as the sensitivity analysis further adjusting for education, smoking and BMI in the multivariable IVW models, were consistent with the main IVW estimates (supplementary Table S7). Repeating the analysis using genetic associations with BP unadjusted for BMI yielded univariable (supplementary Table S8) and multivariable (supplementary Table S9) results with a similar interpretation.

## Discussion

Consistent with previous MR studies^18–20^, and trials^21,22^, higher systolic and diastolic BP were associated with higher risk of CVD. Multivariable MR showed that higher systolic BP was associated with higher risk of CVD overall and by subtype. Diastolic BP was nominally associated with coronary artery disease, but not with CVD overall and other subtypes independent of systolic BP. However, both systolic and diastolic BP independently contributed to lifespan. Taken together, our results demonstrate that systolic and diastolic BP are both targets of intervention.

Although we used a study design less susceptible to confounding than conventional observational studies, our findings are broadly consistent with observational studies showing that systolic-diastolic and isolated systolic hypertension are associated with higher risk of CVD, with less conclusive findings for isolated diastolic hypertension^8,9,23,24^. Using genetic variants independently predicting BP which were stringently selected by LD independence and external replicability in this MR study, we found that lifetime exposure to higher diastolic BP was associated with shorter lifespan and possibly higher risk of coronary artery disease, even after accounting for effects via systolic BP, which are less consistent with previous multivariable MR analyses^12,13^. Here, the multivariate F statistics for the genetic instruments for systolic and diastolic BP were greater than the rule-of-thumb value of 10, circumventing weak instrument bias possibly towards the null in a previous MR^12,25^. We extensively validated our multivariable MR findings. Other than removing SNPs associated with confounders of BP and CVD/lifespan^13^, we used other multivariable MR methods, including multivariable weighted median and MR-PRESSO, we also used genetic associations unadjusted for BMI to circumvent selection bias due to conditioning on heritable traits^26^. Our findings therefore provide additional confirmation of the harmful effects of diastolic BP independent of systolic BP. Notably, effects of diastolic BP on CVD were more substantially attenuated than those of systolic BP when considering systolic and diastolic BP together. However, we observed independent effects of diastolic BP reducing lifespan proxied by parental attained age inclusive of deaths occurring at younger ages. On one hand, these results may suggest that the total effects of diastolic BP can be largely attributed to systolic BP given systolic and diastolic BP are highly correlated phenotypically^27^ and genetically^28^. On the other hand, diastolic BP was less consistently associated with CVD by subtype as it was not associated with ischaemic stroke, heart failure or atrial fibrillation. We cannot exclude the possibility of selection bias attenuating estimates for diastolic BP because GWAS are a non-random subset of their underlying birth cohorts who survive long enough to be included^29^, thus necessitating the inclusion of lifespan phenotypes less affected by this issue. Isolated diastolic hypertension is more common in early life^30^, so the possibility remains that higher diastolic BP precludes survival to recruitment more strongly than higher systolic BP, meaning that estimates for diastolic BP on the CVDs that occur later in life, such as ischaemic stroke, heart failure and atrial fibrillation^31^, are more artefactually attenuated than those for systolic BP. Observationally, in a young sample, isolated diastolic and isolated systolic hypertension had similar contributions to major CVD events^32^. Systolic and diastolic BP had similar total and direct effects on lifespan, consistent with a previous MR study^14^, which again suggests the possibility that estimates for diastolic BP are artefactually attenuated by diastolic BP having a greater effect than systolic BP on mortality at younger ages from other causes of death^33^.

Some limitations of this study should be mentioned. The validity of MR relies on the three major instrumental variable assumptions. First, the genetic instruments should be associated with the exposure, here we used SNPs strongly predicting BP with external replication to minimise false positives. Second, the genetic variants are not associated with confounders of the exposure and outcome. Third, the genetic variants are associated with the outcome only through the exposure. Although we could not definitively test the second and third assumptions, we used MR-Egger and conducted a sensitivity analysis adjusting for major confounders of BP and CVD, we did not find strong evidence of directional pleiotropy. Multivariable MR additionally requires no perfect collinearity between the genetic associations with the exposures^34^. We included genetic variants independently predicting systolic and diastolic BP, with the conditional instrument strength well above the rule-of-thumb value, so the multivariable MR estimates are unlikely to be explained by multicollinearity^17^. Fourth, this was a two-sample MR study using summary statistics, meaning we could only assess linear relations of BP with CVD and lifespan, and our findings could not be used to inform the optimal BP levels given the long-debated J- (or U-) shaped relationship of BP with mortality and morbidity^35^. However, confounding by poor health may give rise to the J-curve^36^, which is increasingly refuted^37^, and could be further tested using individual-level data^38^. We also could not assess differences by sex, because relevant sex-specific summary statistics are not available. Fifth, the validity of MR maybe influenced by canalisation, where genetic variation can be eliminated by compensatory mechanisms during development^39^. However, canalisation biases the effect estimates towards the null^10^, which does not explain the positive associations here. Sixth, the UK Biobank was used for the exposures and some of the outcomes, which might result in bias in the direction of the observational association. However, we used strong genetic instruments for BP so bias due to weak instruments is unlikely^40^. The SNPs also had high I^2^ statistics, so bias due to confounding between exposure and outcome in overlapping samples is unlikely for the IVW and weighed median estimates, although some bias inflating the MR-Egger estimates is possible given a higher I^2^ is desired, so the MR-Egger estimates should be interpreted with caution^41^. Seventh, we constructed major CVD events by combining summary statistics of several potentially correlated outcomes, which could result in underestimation of the standard error, but at the same time takes into account the effect of a genetic variant on several phenotypes of interest. To correct for this possibility, we inflated the standard error^42^, we also investigated several common CVD subtypes as validation. Eighth, the BP GWAS adjusted for BMI to control for confounding, which could be a source of selection bias^43^, resulting in potential reversal of MR estimate direction due to unobserved common causes of BMI and exposure/outcome^44^, or common causes of survival and outcome^45^. However, adjusting for potential common causes, such as education, smoking and BMI, did not change the estimates. A previous MR using genetic predictors of BP without adjustment for BMI showed similar univariable MR estimates for BP on stroke^19^. Our sensitivity analysis provides similar reassurance. Ninth, the association for the direct effect of diastolic BP on coronary artery disease only reached the nominal but not Bonferroni-corrected significance threshold, suggesting insufficient strength of evidence. However, we found strong evidence of a direct effect of diastolic BP on lifespan, future studies using larger sample sizes for coronary artery disease should further elucidate this finding. Finally, although most of the outcome GWASs were conducted in European-descent individuals, the CARDIoGRAMplusC4D consortium consisted of participants from multiple ancestral groups, hence confounding by population structure is possible^46^. However, most of the participants were of European descent (77%), and some of the non-European studies adjusted for genetic principal components to correct for population structure^47^, we also included the UK Biobank estimates to increase the precision, the pooled estimate was 93% European so any bias due to population structure is likely to be minimal.

From a population health perspective, the findings in the present study suggest that within the usual population range, the “lower the better” concept for systolic BP is also relevant to diastolic BP^48^, and our study provides some genetic validation supporting the benefits of a lower diastolic BP threshold for hypertension^49^, which may perhaps motivate changes in other existing guidelines^50^. In conjunction with the 2017 ACC/AHA guidelines, the inclusion of more individuals for BP management and a lower BP target, regardless of systolic or diastolic BP, may produce enormous health benefits in the face of population ageing.

In conclusion, this present MR study showed higher systolic and diastolic BP to be independently associated with higher risk of coronary artery disease and shorter lifespan. Our findings provide supporting evidence of lower systolic and diastolic BP targets in the 2017 ACC/AHA guidelines.

## Methods

### Genetic instruments for blood pressure

We obtained strong (P < 5 × 10^–8^), independent (r^2^ < 0.001) and externally replicated single nucleotide polymorphisms (SNPs) from the genome-wide association studies (GWAS) for BP traits by Evangelou et al.^28^ which provided summary statistics on ~ 757,601 individuals of European ancestry (grasp.nhlbi.nih.gov/FullResults.aspx), with a mean age of 56.0 years, of which 54.7% were women. The summary statistics were generated by fixed-effects inverse-variance weighted (IVW) meta-analysis of the International Consortium for Blood Pressure (ICBP) and the UK Biobank. Both the ICBP and the UK Biobank GWASs adjusted for age, age^2^, sex and body mass index (BMI), and included study-level genomic control accounting for population structure, as well as correction for observed BP based on hypertension medication status by adding 15 and 10 mmHg for systolic and diastolic BP, respectively. Palindromic SNPs with an effect allele frequency > 0.42 and < 0.58, which are difficult to align, were replaced with proxies (r^2^ ≥ 0.8 obtained from LDlink^51^) wherever available. The pooled mean (standard deviation (SD)) systolic and diastolic BP were 138.4 (20.1) and 82.8 (11.2) mmHg, respectively^28^.

### Genetic associations with CVD and its subtypes

CVD was considered as major CVD events defined as a composite of coronary artery disease, stroke (intracranial haemorrhage and ischaemic stroke) and heart failure, similar to SPRINT^22^. Genetic associations with CVD (Table 1) were obtained from the UK Biobank (pan.ukbb.broadinstitute.org) participants of European ancestry on ~ 420,531 individuals^52^. To obtain the composite CVD outcome, we combined the summary statistics for each of the four CVD subtypes from the UK Biobank, which reflect their relative proportion in the population, using fixed-effects IVW meta-analysis. Given these subtypes are likely correlated, we applied genomic control by inflating the standard error using the square root of the linkage disequilibrium (LD) score regression intercept (1.337)^53,54^. LD score regression is a linear regression of SNP association chi-square statistic on SNP LD score, a departure from unity of the intercept indicates bias in the effect sizes due to population structure. We estimated the LD score regression intercept using LD Hub^55^.

**Table 1:** Genome-wide association studies for the CVD, its subtypes and longevity.

Outcome	Consortium/cohort (phecode)	Sample size (no. of cases)	% UK Biobank participants	Covariate adjustment^a^
Coronary artery disease	UK Biobank (411)	419,724 (37,672)	100	Age, sex, age*sex, age^2^, age^2^ * sex, and the first 10 PCs
Coronary artery disease	CARDIoGRAMplusC4D	184,305 (60,801)	0	Age, sex and up to 10 PCs in some non-European samples
Intracranial haemorrhage	UK Biobank (430)	410,070 (2,437)	100	Age, sex, age*sex, age^2^, age^2^ * sex, and the first 10 PCs
Ischaemic stroke	UK Biobank (433)	418,936 (11,303)	100	Age, sex, age*sex, age^2^, age^2^ * sex, and the first 10 PCs
Ischaemic stroke	MEGASTROKE	514,791 (60,341)	0	Age, sex and up to 10 PCs
Heart failure	HERMES	977,323 (47,309)	40	Age, sex and up to 12 PCs
Heart failure	UK Biobank (428.2)	419,557 (7,451)	100	Age, sex, age*sex, age^2^, age^2^ * sex, and the first 10 PCs
Atrial fibrillation	Nielsen et al. 2018	1,030,836 (60,620)	38	Age/birth year, sex and up to 4 PCs
Combined parental attained age	UK Biobank Pilling et al. 2017	389,166	100	Offspring age, sex, assessment centre, array type and the first 5 PCs

*CARDIoGRAMplusC4D* Coronary Artery Disease Genome-wide Replication and Meta-analysis plus the Coronary Artery Disease Genetics, *CVD* cardiovascular disease, *HERMES* Heart Failure Molecular Epidemiology for Therapeutic Targets, *PC* principal component.

^a^Some sub-studies of the genetic consortia also adjusted for other study-specific covariates.

We included major CVD subtypes from the largest and most recent publicly available genetic consortia for validation, including of coronary artery disease^56^, ischaemic stroke^57^, heart failure^58^ and atrial fibrillation^59^. Where these genetic consortia did not overlap with the UK Biobank, we included the MR estimates from the UK Biobank to improve the precision of our estimates. We did not present the MR estimates for intracranial haemorrhage because no large GWAS was available for meaningful interpretation^60^, nor has it accrued a substantial number of cases in the UK Biobank. We considered atrial fibrillation for CVD subtype validation because it was studied in a secondary analysis of the SPRINT results^61^.

### Genetic associations with lifespan

We obtained genetic associations with combined parental attained age, as a proxy of longevity, from the UK Biobank participants of British ancestry^62^. We used parental longevity as a proxy of longevity because it enables use of a larger sample, and parental longevity is correlated with individual longevity phenotypically and genetically^63,64^. Participants provided the current age, or the age at death of their parents. The mean parental age at death (SD) was 78.6 (9.8) for mothers and 72.2 (11.1) for fathers, and 151.6 (15.0) years for both parents combined^62^. The attained age was standardised to the sex-specific mean value before being combined to account for the generally longer lifespan of women.

### Statistical analysis

We assessed the strength of the genetic instruments using the F-statistic, approximated by the squared SNP-exposure association divided by the variance of this association. An F-statistic < 10 indicates a potentially weak instrument^65^. We conducted power calculations, based on the approximation that the sample size needed in an MR study is approximately the sample size for an conventional observational study divided by the proportion of variance in exposure explained by the genetic instrument^66^. SNPs instrumenting BP but unavailable for an outcome were replaced by proxies (r^2^ ≥ 0.8) wherever available. SNPs were aligned on the same effect allele for exposure and outcome. We used the MR Steiger test of directionality to assess whether the selected SNPs explained a larger proportion of the phenotypic variance of BP, than of the outcomes^67^.

To estimate the total effects of systolic and diastolic BP (per SD increment) we used univariable MR, specifically IVW with multiplicative random effects to combine the SNP-specific Wald estimates, obtained by dividing the SNP-outcome association by the SNP-exposure association. Given, the IVW estimates with multiplicative random effects assumes balanced pleiotropy^68^, we conducted sensitivity analyses using the weighted median, MR-Egger and MR pleiotropy residual sum and outlier (MR-PRESSO). The weighted median estimates are robust to directional genetic pleiotropy as long as more than 50% of the weight comes from valid SNPs^69^. The MR-Egger estimates are robust to directional genetic pleiotropy even if all SNPs are invalid instruments, provided the Instrument Strength Independent of Direct Effect (InSIDE) assumption is satisfied. A non-zero MR-Egger intercept indicates potential directional pleiotropy and possible bias in the IVW estimates^70^. We assessed the variability of instrument strength using the I^2^ statistic. An I^2^ < 90% suggests violation of the no measurement error (NOME) assumption and an invalid MR-Egger estimate^65^. An I^2^ ≥ 97% suggests bias due to overlapping samples for exposure and outcome is minimal^41^. MR-PRESSO statistically detects horizontal genetic pleiotropy and produces corrected estimates after removal of outliers^71^. We conducted a supplementary analysis using pulse pressure as the exposure given a widening pulse pressure reflects increasing difference between systolic and diastolic BP. We used Phenoscanner to identify which of the SNPs predicting BP was also strongly (P < 5 × 10^–8^) associated with education, smoking or BMI^72^, and we conducted a sensitivity analysis excluding such SNPs.

To estimate direct effect of systolic and diastolic BP we used multivariable MR of independent (r^2^ < 0.001) SNPs instrumenting systolic or diastolic BP obtained using the “clump_data” function of the “TwoSampleMR” R package, based on the European 1000 Genomes catalog, and the strength of their associations with systolic or diastolic BP, retaining the lower P-value when a SNP instrumented both BPs. We used multivariable IVW^16^, weighted median^73^, MR-Egger^74^ and MR-PRESSO^71^ to estimate the direct effect. We estimated the Sanderson–Windmeijer multivariate F-statistic to assess the conditional instrument strength, as well as the modified Q statistic as a measure of instrument pleiotropy^17^, taking into account the phenotypic correlation between systolic and diastolic BP (Pearson correlation 0.69 in the UK Biobank) to estimate the covariance of SNP-exposure associations^25,75^. To assess whether the multivariable MR results were robust to horizontal pleiotropy^45^, we included a sensitivity analysis which further adjusted for major confounders of BP and CVD, namely education (number of years)^76^, smoking (ever or never)^42^ and BMI^77^ by including their genetic associations in the multivariable IVW model. We included BMI in the multivariable MR model because genetic associations with the outcomes were independently estimated and did not account for the pleiotropic effects via BMI, despite the BP GWAS having already adjusted for BMI.

As the BP GWAS adjusted for BMI, potentially resulting in selection bias of the genetic associations with BP via common causes of BP and BMI^26^, we repeated the univariable and multivariable analyses using genetic associations with BP unadjusted for BMI extracted from the UK Biobank. We presented the estimates as per SD increment in the main text for direct comparison of systolic and diastolic BP, and, for interpretability, as per 10 (systolic BP) and 5 (diastolic BP) mmHg in the supplementary materials. With consideration of multiple testing and the similar aetiology of the CVDs included in the present study, a Bonferroni-corrected p-value of 0.0125 (0.05/2 BP traits*2 outcome groups) was considered strong evidence whereas a nominal p-value < 0.05 but > 0.0125 was considered suggestive evidence^34^, which adequately controls for multiple testing while avoiding false negatives. We used the R packages “TwoSampleMR” to obtain univariable MR estimates, the “MendelianRandomization” package to obtain multivariable MR estimates, and the “MVMR” package to obtain the Sanderson–Windmeijer multivariate F-statistic and the modified Q statistic. All statistical analyses were conducted using R (version 4.0.1, The R Foundation for Statistical Computing Platform, Vienna, Austria).

### Ethics

This study only used publicly available data. No original data were collected. Ethical approval for each of the studies included in the investigation can be found in the original publications.

## Supplementary Information

Supplementary Information.
